# Preclinical advances in antibodies against N‐terminal Aβ4‐x species for Alzheimer's disease and cerebral amyloid angiopathy

**DOI:** 10.1002/ibra.70016

**Published:** 2026-03-01

**Authors:** Maria Luisa Valle, Donatello Arienzo

**Affiliations:** ^1^ Department of Pathology NYU Grossman School of Medicine New York New York USA; ^2^ Department of Psychiatry San Diego State University San Diego California USA

**Keywords:** Alzheimer's disease (AD), amyloid beta, cerebral amyloid angiopathy, immunotherapy, preclinical studies

## Abstract

Alzheimer's disease (AD) pathogenesis is characterized by the accumulation of amyloid beta (Aβ) deposits in the cerebral parenchyma and vasculature, a condition referred to as cerebral amyloid angiopathy (CAA). Besides the full‐length form, Aβ deposits showed a highly heterogenous composition due to the action of different proteolytic enzymes. N‐terminal Aβ peptides have shown higher aggregation propensity and toxicity compared to the other truncated forms, with species starting at residue phenylalanine 4 (Aβ4‐x) being more neurotoxic than the others. Thus, Aβ4‐x species have drawn attention in AD pathogenesis, potentially offering novel therapeutic targets to halt or reverse disease progression. Antibodies targeting specifically Aβ4‐x species were designed with the aim of preventing their aggregation and promoting their clearance counterbalancing their neurotoxic effect. This work provides an update on monoclonal and polyclonal antibodies developed to specifically target Aβ4‐x species in AD and CAA preclinical studies (in vitro and in vivo models).

## Graphical abstract

This review provides an update on monoclonal and polyclonal antibodies developed to specifically target amyloid beta (Aβ) truncated species starting at residue phenylalanine 4 (Aβ4‐x) in Alzheimer's disease and cerebral amyloid angiopathy preclinical studies (in vitro and in vivo models).

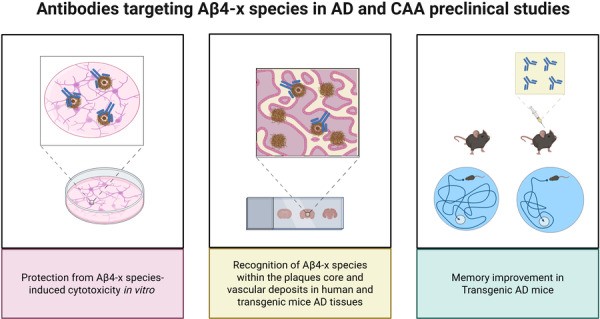

## 1 INTRODUCTION

Alzheimer's disease (AD) is the most common form of dementia, counting about 55 million patients worldwide, which are estimated to increase to 152 million in 2050.^1^ AD is characterized by the accumulation of amyloid beta (Aβ) plaques in the human brain parenchyma and within cerebral vessels (known as cerebral amyloid angiopathy [CAA]), hyperphosphorylated neurofibrillary tangles and synapse loss. The pivotal role of Aβ in AD pathogenesis has been strongly supported through the decades via the amyloid cascade hypothesis as well as the genetic component.^2^, ^3^, ^4^, ^5^ Beside the well‐known formation of Aβ1‐40 and Aβ1‐42 species, several shorter truncated species at the amino (N)‐ or carboxyl (C)‐ terminal have been reported over the years.^6^, ^7^, ^8^, ^9^, ^10^ In particular, N‐terminal species were reported to be highly toxic and have higher aggregation propensity with species starting at residue phenylalanine (Phe) 4 (Aβ4‐x) being more neurotoxic than the others.^11^ Over the last two decades, different research groups have developed monoclonal and polyclonal antibodies targeting Aβ species starting at position 4, unraveling interesting findings about the pathophysiology of AD and vascular dementia. This work provides an update on the monoclonal and polyclonal antibodies developed to specifically target N‐terminal Aβ4‐x species in preclinical studies (in vitro and in vivo models). A detailed description of N‐ and C‐terminal Aβ truncated species or immunotherapies targeting Aβ species employed in clinical studies has been described elsewhere.^12^, ^13^, ^14^, ^15^, ^16^, ^17^, ^18^

## 2 METHODOLOGY

A comprehensive search for original research articles focusing on monoclonal and polyclonal antibodies developed to specifically target N‐terminal Aβ4‐x species in preclinical studies was performed in PubMed by searching “preclinical studies OR antibodies AND truncated amyloid beta AND N‐terminal species OR Aβ4‐x”. Only studies written in English were eligible for inclusion and published between 1990 and 2025 were considered.

## 3 PROPERTIES OF AΒ4‐X SPECIES

N‐terminal truncated Aβ peptides, in particular species beginning at Phe 4, were first described in 1985 and have been reported in patients with AD, Down's syndrome, and vascular dementia.^6^, ^7^, ^19^, ^20^, ^21^ Among them, Aβ4‐42 was the first N‐truncated peptide discovered.^7^ Sergeant et al. extracted aggregated amyloid species from human brain tissues using formic acid and two‐dimensional gel electrophoresis followed by mass spectrometry (MS) for identification. The MS analysis showed that the majority of amyloid species aggregated in the early AD stages were amino‐truncated, and western blot analysis indicated that they represented more than 60% of all amyloid species in amyloid deposits.^22^ However, it is important to highlight the experimental and methodological limitations of the study, as more heterogeneous signatures were reported over the years.^23^, ^24^ Lewis et al. described Aβ4‐42 species particularly abundant in AD and CAA patients while Portelius et al. detected Aβ1‐40, Aβ1‐42 and Aβ4‐42 within the hippocampus and cortex of AD patients using immunoprecipitation coupled with mass spectrometry (IP/MS).^19^, ^25^ A series of peptides with truncated N‐terminal (starting at position 4,8,12 and 17) and C‐terminal ending at position 40 or 42 was synthesized by Pike et al. who then used circular dichroism (CD), transmission electron microscopy (TEM) and in vitro models to analyze their properties.^11^ The authors reported that peptides with the N‐terminal deletion showed a predominant β‐sheet conformation, enhanced aggregation, and higher toxicity when added to hippocampal neurons. Compared to Aβ1‐42, the N‐terminal deletions in Αβ4‐42 enhanced amyloid aggregation properties suggesting the critical role of the N‐truncated species.^11^, ^26^ Later, Parodi‐Rullan et al. challenged cerebral microvascular endothelial cells with different C‐ and N‐terminal truncated Αβ species to study how they would affect the blood‐brain barrier (BBB) permeability, angiogenesis, and microvascular dysfunction in CAA.^27^ Among the peptides, Aβ4‐40 and Aβ4‐42 both affected angiogenesis, with Aβ4‐42 being the most potent angiogenic inhibitor. Aβ4‐42, but not Aβ4‐40, also showed a detrimental effect on BBB permeability.^27^

Due to the N‐terminal deletion, Aβ4‐x species are characterized by distinct biophysical properties compared to full‐length or other post‐translationally modified amyloid species. The removal of the charged N‐terminal residues induces a predominant β‐sheet conformation and higher aggregation propensity in Aβ4‐x species, resulting in increased neurotoxicity than the full‐length counterparts.^11^, ^26^, ^28^ Thioflavin T binding assay showed high binding to Aβ1‐42 and Aβ4‐42, while intermediate activity to Aβ1‐40 and Aβ4‐40, although the truncated peptide showed a higher activity than its full‐length counterpart.^28^ These properties also suggest that those species may have a key role in the initiation of the pathological deposition of Aβ. Aβ4‐x species is abundant in vascular amyloid deposits and CAA due to its low solubility and enhanced aggregation, resulting into poor clearance and accumulation in blood vessel walls.^28^, ^29^ Pyroglutamate‐modified Aβ3‐x species (AβpE3‐x) is among the main Aβ species identified in human AD brains, representing 25% of the senile plaques.^25^, ^30^ The change in the species' charge due to the modification results in an enhanced hydrophobicity and accelerated aggregation kinetics compared to full‐length amyloid.^31^ Like Aβ4‐x species, AβpE3‐x has a higher tendency to form β‐sheet structures, was detected within the plaques' core and showed a comparable toxicity.^32^ Based on the modified amyloid hypothesis suggesting that Αβ accumulation is intraneuronal prior to extracellular, Antonios et al. reported that intraneuronal Aβ4‐x accumulation preceded AβpE3‐x in 5XFAD mice prior to plaque formation.^32^, ^33^

As regards in vivo studies, wild type mice intraventricularly injected with the Aβ4‐38, Aβ4‐40 and Αβ4‐42 species experienced working memory deficits when performing Y‐maze test.^26^ Amyloid precursor protein (APP)/presenilin 1 (PS1) knock‐in (KI) mice, expressing transgenic human mutant APP751 (including the Swedish and London mutations) on a murine KI PS1 carrying mutations M233T/L235P.^34^, ^35^ The model, characterized by high abundance of Αβ4‐42 and N‐truncated species (in particular Αβ4‐42), showed neuronal loss within hippocampus, frontal cortex and cholinergic nuclei as well deficits in working memory.^35^, ^36^ In 2013, Bouter et al. developed a transgenic Αβ4‐42 (Tg4‐42) mouse model to study in vivo chronic exposure to the Αβ4‐42. Tg4‐42 mice contained human Αβ4‐42 fused to the murine thyrotropin‐releasing hormone signal peptide under the control of the Thy‐1 promoter. From 2 months of age, the model showed a strong immunoreactivity in the hippocampal CA1 region, while during aging, Tg4‐42 mice experienced severe neuronal loss as well as spatial memory deficits.^26^ To our knowledge, there are limited studies investigating if the genetic background of the cited murine strains may influence Aβ4‐x species abundance. Aβ4‐x and Aβ1‐x species were detected in the intraneuronal compartment of cortical neurons in 1‐year‐old 5XFAD mice, while APP/PS1 KI mice exhibited differential intraneuronal and extraneuronal Aβ accumulation within brain regions, but Aβ4‐x species were not investigated.^37^, ^38^

## 4 FORMATION OF AΒ4‐X SPECIES

Several enzymes have been proposed to mediate the cleavage of full‐length Aβ to N‐terminal truncated species over the years. Initially, it has been hypothesized that the formation of Aβ4‐x species may occur after cleavage of glutamate at position 3 of Aβ. This may result after cleavage by exopeptidases before its cyclization, or removal of the pyroglutamate‐modified (pE3) residue once formed by the acidic protease aminopeptidase A.^39^ In the alternative, pyroglutamyl peptidases I and II may attack the pE3 residue.^40^ Another hypothesis included non‐enzymatic generation by spontaneous decomposition of full‐length Aβ peptides. The phenomenon has been reported for the generation of amino and carboxyl terminal fragments and Aβ3‐x species as well, but there is no evidence that it can occur to Aβ4‐x species.^41^, ^42^ Walter et al. also described the involvement of the secreted ADAM metallopeptidase with thrombospondin type 1 motif 4 (ADAMTS4) in Aβ4‐x species generation, identifying a recognition site within the Aβ sequence.^43^

## 5 ALTERED BRAIN CLEARANCE OF AΒ4‐X SPECIES

Brain clearance mechanisms have been recognized as key factors in regulating the delicate balance between Aβ production, accumulation, and efflux within the brain.^44^, ^45^, ^46^ The zinc metalloprotease neprilysin was identified as an Aβ‐degrading enzyme in transgenic mice, increasing the interest in investigating Aβ catabolism.^47^ Interestingly, besides Aβ1‐40 and Aβ1‐42, Aβ4‐40 and Aβ4‐42 species were also reported among neprilysin substrates.^48^ Other described Aβ‐degrading enzymes include angiotensin‐converting enzyme (ACE), metalloproteases, insulin‐degrading enzyme (IDE), cathepsin B and D, and β‐amyloid cleaving enzyme 1 (BACE1).^49^, ^50^, ^51^, ^52^, ^53^, ^54^ In addition to Aβ degrading enzymes, other cellular mechanisms have been hypothesized, including transport to cerebrospinal fluid (CSF), perivascular drainage, phagocytosis mediated by glial cells and BBB clearance, as most studied mechanism.^46^, ^55^, ^56^ Reduction in brain clearance plays a pivotal role in AD pathogenesis, especially in elderly patients.^44^ By analysis of CSF of wild‐type mice injected with monomeric Aβ1−40, McIntee et al. showed that Αβ physiological removal is fast (Aβ degradation products were detected after only 5 min post‐injection) and involves local proteolytic degradation with formation of C‐terminal species.^57^ Other studies also described Aβ4‐x and other amino terminally truncated amyloid forms in CSF, suggesting their ability to cross the BBB.^58^, ^59^ Rostagno et al. injected [125^I^]‐labeled Aβ peptides (Aβ1‐34, Aβ1‐40, Aβ1‐42, Aβ4‐40, Aβ4‐42) into C57BL/6 mice to evaluate their respective efflux from the brain.^28^ Interestingly, 80% of Aβ species were cleared within 60 min with N‐terminal species showing higher brain retention compared to C‐terminal peptides that had a faster elimination. Both Aβ4‐40 and Aβ4‐42 showed enhanced retention than the respective full‐length peptides. These findings further supported the hypothesis that the rate of brain clearance is inversely correlated to the oligomerization tendency of the amyloid species. Whether this is a consequence of their increased insolubility due to their structure or depends on a decreased binding efficiency to brain efflux transporters is still unclear.

## 6 RELEVANCE OF AΒ4‐X SPECIES IN CAA

More than 90% of AD cases manifest cerebrovascular deposition of amyloid, also known as CAA. CAA has been associated with focal ischemia, cerebral hemorrhage, and neurovascular dysfunction and is characterized by Aβ1‐40 as the main Aβ isoform contributing to the vascular Aβ deposits.^60^ Depending on the topographical localization of the Aβ deposition, CAA can be classified into Type 1 CAA (deposition on cortical capillaries, leptomeningeal and cortical arteries) or the more common Type 2 CAA (leptomeningeal and cortical vessels).^61^ In both types, the amyloid deposition around cerebral vessels progressively affects cerebral blood flow, BBB permeability, and brain clearance, impacting the overall integrity of the neurovascular unit.^62^, ^63^ However, vascular amyloid deposits have proved to be more complex than the Aβ1‐40/Aβ1‐42 dichotomy, as the presence of multiple posttranslational modifications and truncated forms has been characterized. In general, N‐terminal truncated species (including Aβ4‐x species) are enriched in CAA due to their low solubility and enhanced aggregation, which leads to poor clearance and accumulation in blood vessel walls.^28^, ^29^ Aβ4‐x species are less soluble than full‐length forms and require more stringent extraction conditions (like 2% sodium dodecyl sulfate or 70–99% formic acid) to be detected.^29^

The majority of preclinical CAA models consist of transgenic mouse models in which CAA develops secondary to AD‐related plaque pathology with the exception of APP Dutch and Tg‐SwDI mice that were primarily designed to study CAA.^60^, ^64^, ^65^ APP Dutch mice overexpress the E693Q‐mutated human APP_751_, developing vascular amyloid deposits at a late age (22–25 months) with rare parenchymal plaques.^65^ Tg‐SwDI mice express human APP_770_ containing the Swedish double mutation (K670N/M671L) and the Dutch and Iowa (D694N) mutations, thus manifesting CAA starting at 3 months of age, with more than 50% of microvasculature affected at 12 months. A complete description of preclinical CAA models has been reported by Jakel et al.^64^

Vascular integrity is often compromised by cerebrovascular amyloid deposition, leading to amyloid‐related imaging abnormalities (ARIA) in the form of vasogenic edema (ARIA‐E) or microhemorrhages (ARIA‐H) as adverse effects of anti‐amyloid immunotherapies. Although the mechanisms causing ARIA are still under investigation, it has been hypothesized that they may be related to the antibodies' interaction with vascular amyloid in CAA, triggering a cascade of events involving inflammation, complement activation, and increased vessel permeability. Recently, an increased expression and activity of matrix metalloproteinases (MMPs), like MMP9 and MMP3, were reported in CAA.^66^ Due to their involvement in basement membranes and tight junction proteins degradation, MMPs activation could lead to edema and hemorrhage. Although CAA is often asymptomatic, it can be detected via computed tomography (CT) and magnetic resonance imaging according to the modified Boston criteria, while amyloid positron emission tomography imaging showed poor accuracy due to the inability to differentiate between vascular and parenchymal amyloid.^67^, ^68^ Potential biomarkers include Aβ40, Aβ40/42, phosphorylated‐tau217, neurofilament light chain (NfL), glial fibrillary acidic protein, secreted phosphoprotein 1, placental growth factor, triggering receptor expressed on myeloid cells 2 (TREM2), which have been discussed in detail by Sin et al.^69^

## 7 ANTIBODIES TARGETING AΒ4‐X SPECIES IN PRECLINICAL STUDIES

**Figure 1:**
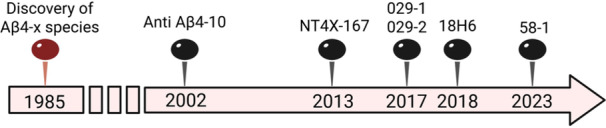
Chronological list of antibodies targeting Aβ4‐x species in preclinical studies. Antibody NT4X‐167 has also been reported to recognize AβpE3‐x species.

In the past decades, considerable attention was given to immunization against Aβ as a vaccination strategy. One approach involved the administration of antibodies against Αβ species via intravenous, subcutaneous, or intraperitoneal routes (known as passive immunization).^70^, ^71^, ^72^ Other AD studies over the decades have exploited Αβ peptides as antigens in a vaccine to elicit an immune response (active immunization) involving activation of B and T cells with antibodies production in the recipient. AN1792 was the first AD vaccine tested in a clinical study using Αβ1‐42 as antigen.^72^, ^73^ Fewer groups developed immunization strategies using Aβ4‐x species, highlighting that they may represent better antigen candidates than the full‐length counterparts due to their abundance, increased toxicity, aggregation, and less adverse effects. The main findings from their in vitro and in vivo studies are described in this section and summarized in Table 1. Figure 1 provides a chronological list of reported antibodies targeting Aβ4‐x species in preclinical studies described in this review.

**Table 1:** Summary of characteristics and properties of anti Αβ4‐x species in in vitro and in vivo studies.

Antibody name	Antibody anti Aβ 4‐10	NT4X‐167	029‐1 and 029‐2	18H6	58‐1
**Production**	TgCRND8 mice immunized with protofibrillar Aβ42	Balb‐c mice immunized with unconjugated Aβ4‐40	Guinea pigs immunized with Aβ4‐9 peptide	Mice immunized with Aβ4‐9 peptide coupled with a keyhole limpet hemocyanin (KLH)	Rabbits immunized with Aβ4‐9 peptide
**Antibody Type**	Monoclonal	Monoclonal	Polyclonal	Monoclonal	Polyclonal
**Immunogens used to generate the antibody**	FRHDSGY	Aβ4‐40	FRHDSG	FRHDSG	FRHDSG
**Isotype**	IgG2b	IgG2b	Not reported	IgG1k	Not reported
**Specificity for Aβ4‐x species**	Aβ4‐10	Aβ4‐x	Aβ4‐x	Aβ4‐x (Aβ4‐40 and Aβ4‐42)	Aβ4‐x (Aβ4‐40)
**Epitope promiscuity**	Not reported	Yes, Aβ3‐x	Not reported	Not reported	Not reported
**Validated applications**	*IHC, WB*	*ELISA, IHC, WB, IP/MS*	*ELISA, IHC, WB*	*IHC, WB, IP/MS*	*IHC, WB*
**Models tested (ms= months)**	TgCRND8 mice (age not specified)	5XFAD mice (6 ms), Tg4‐42 mice (3,8,12 ms)	5XFAD (5 and 10 ms), APP/PS1KI (8 ms)	APP/PS1 (12 and 17 ms), Tg2576 (20 ms), 5XFAD (2–10 ms), APPLd2 (22–28 ms)	5XFAD mice (6 and 10 ms)
**Main findings from in vitro and *in vivo* studies**	Inhibition of cytotoxicity and fibrillogenesis in PC12 cells challenged with Aβ4‐10 peptides Absence of inflammatory response in TgCRND8 mice Recognition of plaques in AD tissues	Protection from cytotoxicity in primary neurons challenged with Aβ4‐x and Aβ3‐x species Recognition of intraneuronal Aβ in 5XFAD mice Binding to Aβ in plaques but not vessels in sporadic and familial AD cases Memory improvement in Tg4‐42 mice (spatial reference, and working memory)	Recognition of neuritic plaque cores and vascular deposits in sporadic AD cases (029‐2) Binding to plaques in the cortex, subiculum, and thalamus in 5XFAD mice (029‐1 and 029‐2)	Binding within the center of the plaques and vascular Aβ deposits in different Tg mice and sporadic AD tissues Tandem IP/MS using 18H6 prior to 4G8/6E10 antibodies, fully retrieved Aβ4‐40 and Aβ4‐42 in APP/PS1 brain extracts	58‐1 showed binding to Aβ4‐x species in the cortex in 6‐ and 10‐month‐old 5XFAD mice Its immunoreactivity was blocked when pre‐incubated with excess of Aβ4‐40
**References**	[74]	[32, 75]	[76]	[28, 77, 78, 79, 80]	[79, 81]

Abbreviations: AD, Alzheimer's disease; ELISA, enzyme‐linked immunosorbent assay; IP/MS, immunoprecipitation‐mass spectrometry; IHC, immunohistochemistry; WB, Western blotting.

### 7.1 Monoclonal anti‐Aβ4‐10 antibody

One of the first attempts to generate antibodies targeting Aβ4‐x species was reported in 2002 by McLaurin et al.^82^ The group described a selective IgG2b antibody against residues 4–10 of Aβ42 able to inhibit both Aβ fibrillogenesis and cytotoxicity in TgCRND8 as an AD mouse model.^82^ TgCRND8 mice were immunized with protofibrillar Aβ42 aggregates and sera were tested for specificity, confirming the ability to recognize Aβ42 forms as well as mature Αβ plaques (but not diffuse Aβ deposits). As a control, another group was immunized with islet‐associated polypeptide (IAPP) as amyloid peptides not expressed in the central nervous system. Sera from IAPP‐immunized mice did not show any reactivity for amyloid, as expected. Interestingly, when the group analyzed the linear sequence and the conformational epitope of the antibody, they identified the sequence FRHDSGY (Aβ4‐10) as the minimal peptide bound to the antibody. Furthermore, the anti‐Aβ4‐10 sera were able to reduce cytotoxicity and fibrillogenesis in vitro in PC12 cells and recognized Aβ1‐42 in AD brain tissue. Another important aspect of the immunotherapy was the lack of an inflammatory response, in contrast with previous immunization studies.^74^, ^83^ The authors speculated that the lack of an inflammatory reaction was due to the absence of a T cell activating domain within residues 4‐10. This domain was instead present with the C‐terminus of Aβ1‐42 vaccine AN1792, leading to severe adverse effects (such as aseptic meningoencephalitis in 6% of the participants) that ultimately led to the halt of its clinical trial.^84^, ^85^

### 7.2 Monoclonal antibody NT4X‐167

**Figure 2:**
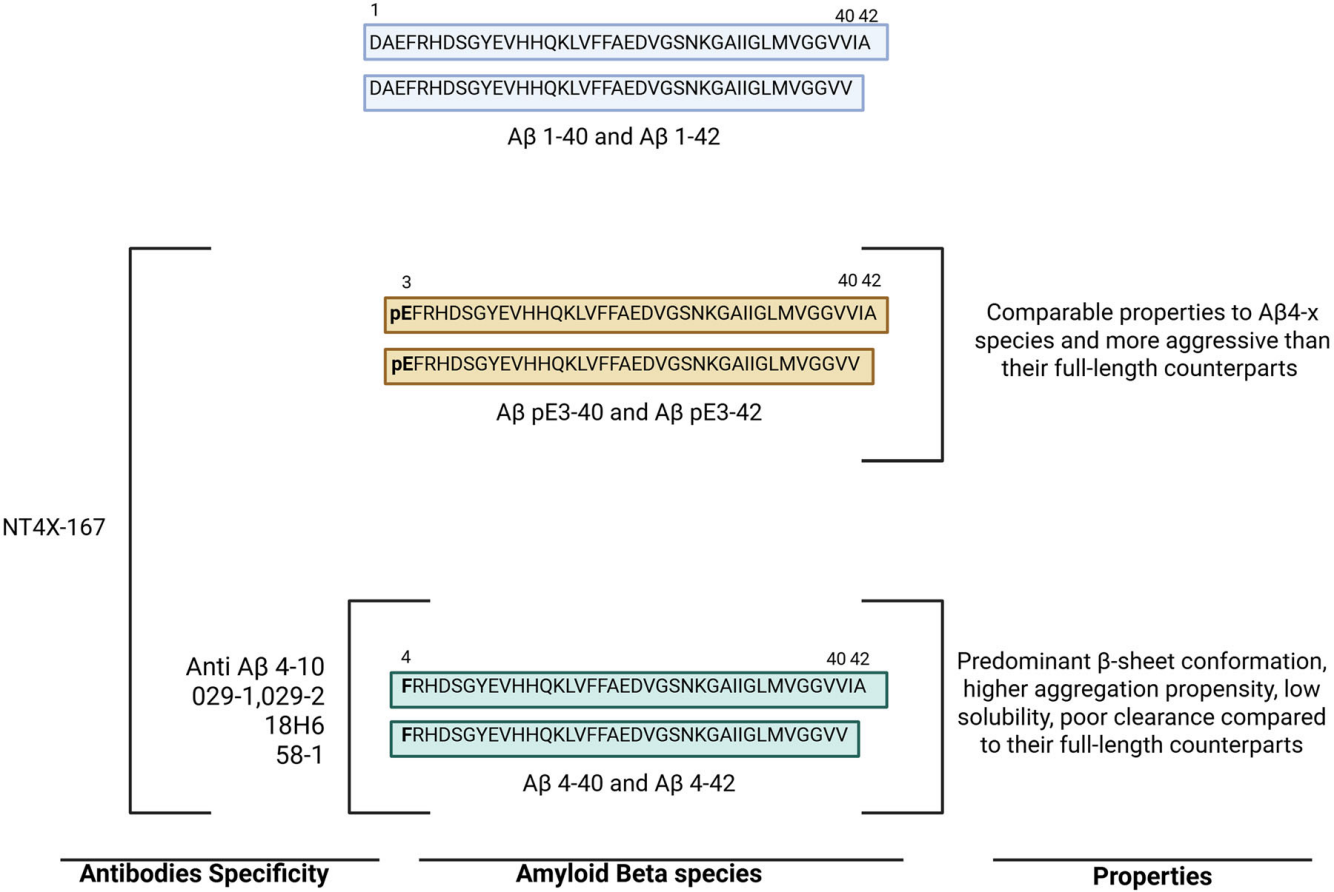
Summary of biophysical properties of Aβ4‐x species compared to full‐length and truncated AβpE3‐x with antibody specificity. (Left panel) Antibodies anti‐Aβ4‐10, 029‐1, 029‐2, 18H6, and 58‐1 recognize N‐ terminal truncated Aβ4‐x species starting at position 4 (F=phenylalanine, central panel). In contrast, antibody NT4X‐167 also recognizes AβpE3‐x species starting at position 3 (pE=pyroglutamate, central panel). Biophysical properties of each N‐truncated species are summarized in the right panel.

The Aβ4‐x immunoreactive antibody NT4X‐167 was firstly reported in 2013 by Antonios et al.^32^ The antibody (IgG2b type) was generated by immunizing Balb/c mice with unconjugated Aβ4‐40. Then lymph nodes were fused with the myeloma cell line P3‐X63‐Ag8 for the generation of hybridoma cells. Via Pepscan ELISA, it was confirmed that the binding site of NT4X‐167 ranged between N‐truncated Αβ2–4 and Αβ4‐x with phenylalanine at position 4 pivotal for the antibody binding. However, when tested under native conditions, the antibody recognized not only Aβ4–40 and Aβ4–42 species but also AβpE3‐40 and AβpE3‐42. A following study, reported in 2015 by the same group, clarified that the NT4X‐167 could recognize N‐terminal Aβ4‐x species as well as pyroglutamate AβpE3‐x species.^86^ Due to this characteristic, NT4X‐167 showed epitope promiscuity, suggesting that the antibody recognizes a shared conformational epitope present in AβpE3‐x and Aβ4‐x species (Table 1 and Figure 2). NT4X‐167 rescued the toxicity induced by both N‐truncated species in primary rat neurons.^32^, ^86^ In addition, immunohistochemical staining of cortical sections of 5XFAD transgenic mice showed that NT4X‐167 recognized intraneuronal Aβ in 6‐week‐old homozygous 5XFAD mice. However, in sporadic and familial AD cases, NT4X‐167 was able to recognize plaques only partially but was positive for CAA staining on vessel walls.^32^ Passive immunization in a 6‐month‐old Tg4‐42 mouse model with NT4X‐167 for 12 weeks significantly rescued neuronal loss, improved spatial reference, and working memory.^86^

### 7.3 Polyclonal antibodies 029‐1 and 029‐2

In 2017, two polyclonal antibodies targeting Αβ4‐x species were described by Wirths et al.^75^ These antibodies were produced by immunizing guinea pigs with the peptide FRHDSG (corresponding to residues 4–9 of the Aβ peptide). The final immune sera obtained and affinity‐purified were named 029‐1 and 029‐2. Both antibodies showed high specificity for Aβ4‐x species, and no cross‐reactivity for Aβ1‐40, Aβ2‐40, Aβ3‐40, AβpE3‐40, and Aβ5‐40 was observed. As further validation, pre‐absorption of 029‐2 with Aβ4‐40 but not Aβ1‐40 peptides was able to block the antibody immunoreactivity for the Aβ4‐x species.^75^ When tested on sporadic AD cases, 029‐2 staining was restricted mainly to neuritic plaque cores and vascular deposits. In a similar trend, 029‐1 and 029‐2 also showed an increased plaque load in the cortex, subiculum, and thalamus of 10‐month‐old 5XFAD mice.^75^

### 7.4 Monoclonal antibody 18H6

In 2018, Cabrera et al. used antibodies against N‐terminal and C‐terminal Aβ truncated species to assess their differential topographic location in transgenic mice and human tissue.^76^ The group described a monoclonal antibody recognizing N‐terminal truncated Aβ species starting at position 4 via mouse immunization with a peptide homologous to amino acids 4–9 of Aβ (sequence FRHDSG). The sequence was coupled with a keyhole limpet hemocyanin (KLH) via an additional cysteine residue. Among the hybridoma cell lines obtained, line 18H6 clone 4 was chosen and used for the studies (IgG1k). A detailed protocol describing culture, characterization, and applications of the 18H6 antibody has been published recently.^77^ The antibody did not show any cross‐reactivity for longer or shorter synthetic homologues or post‐translationally modified by pyroglutamate.^76^, ^78^ When tested on sporadic AD post‐mortem tissues and transgenic mouse strains (APP/PS1, Tg2576, 5XFAD, and APPLd2), 18H6 binding to Aβ4‐x species confirmed their presence in the center of the plaques, mainly in the hippocampal region and within vascular amyloid deposits (co‐localizing with Congo red positive vessels).^76^, ^77^, ^78^, ^79^, ^87^ Co‐staining with thioflavin S also showed the preferential localization of the N‐terminal truncated Aβ4‐x species to the fibrillar lesions. No signal was detected in control cases.^76^ Furthermore, when compared to the humanized monoclonal antibody Bapineuzumab, 18H6 detected the identical extracellular Aβ deposits with comparable sensitivity.^78^, ^80^ In addition, tandem IP/MS analysis using first 18H6 to enrich the precipitate, followed by the mix of 4G8 (binding to Aβ17‐24) and 6E10 (binding to Aβ1‐16) antibodies, fully retrieved Aβ4‐40 and Aβ4‐42 from formic acid APP/PS1 mouse extracts.^76^ These findings support a higher brain retention and poor clearance characteristics of the Aβ4‐x species.^28^

### 7.5 Polyclonal antibody 58‐1

In 2023, Bader et al. tested 18H6 together with a novel rabbit polyclonal antibody, named 58‐1, generated by immunization of rabbits with a peptide comprising amino acids 4–9 of the Aβ sequence.^87^ The selectivity of the antibody 58‐1 for Aβ4‐x species was evaluated via dot‐blot immunoassay for different N‐terminal Aβ variants (Aβ1‐40, Aβ2‐40, Aβ3‐40, Aβ4‐40, and Aβ5‐40), confirming its selectivity exclusively for Aβ4‐40 among the tested synthetic peptides.^87^ In addition, 58‐1 showed binding to Aβ4‐x species in the cortex in 6‐ and 10‐month‐old 5XFAD mice, and its immunoreactivity was blocked when pre‐incubated with an excess of Aβ4‐40.^87^ In 2025, Talucci et al. further characterized the antibody's epitope, showing the need for a free Phe4‐residue at the N‐terminus for its binding.^88^

## 8 OTHER TRUNCATED AΒ SPECIES IN PRECLINICAL STUDIES

Besides Aβ4‐x species, it is important to mention the involvement of other N‐terminal truncated species in AD and CAA preclinical studies, including Aβ2‐x, Aβ3‐x, and Aβ5‐x. A detailed description of immunotherapies targeting Aβ species employed in clinical studies can be found elsewhere.^12^, ^13^, ^14^

### 8.1 Aβ2‐x

Elevated levels of Aβ2‐40 species (together with Aβ1‐40 and Aβ1‐38) were described in the brains of AD patients with CAA, while in the absence of CAA 1‐42, Aβ2‐42, Aβ3pE‐42, and Aβ11pE‐42 were detected.^81^, ^89^ The production of Aβ2‐x species (starting at Alanine 2) may derive from the combined action of BACE1 followed by aminopeptidase A, although recently the involvement of the metalloprotease meprin β was reported.^15^, ^90^ In support of this hypothesis, Armbrust et al. generated a novel meprin β‐overexpressing mouse model, which showed elevated amyloidogenic APP processing as well as AD‐related behavior changes (hyperlocomotion and deficits in spatial memory).^91^ To detect Aβ2‐x species, a novel neo‐epitope‐specific antibody was designed and tested via immunohistochemistry and western blot, proving its specificity.^91^ In addition, Savastano et al. described another preclinical antibody targeting Aβ2‐x species (pAb77) that was generated against the Aβ2‐14 epitope.^89^ The antibody was able to detect Aβ2‐x species and its distribution in AD cases as well as in vivo (AβPP/PS1KI and 5XFAD transgenic mice). Interestingly, AD cases showed abundant CAA and less plaque pathology, while extracellular amyloid deposits and minor CAA staining were detected in transgenic models.^89^ A similar finding was recently reported by Kasri et al., who compared Aβ and tau pathologies in postmortem brains of patients with APP duplications (APPdup) and Down syndrome using mass spectrometry and imaging. The authors reported an increase in N‐truncated Aβ2‐x peptides APPdup cohort, suggesting that CAA is associated with the accumulation of shorter truncated Aβ species.^21^

### 8.2 AβpE3‐x

AβpE3‐x is among the main Aβ species identified in human AD brains.^25^, ^30^ The production of AβpE3‐x species involves the removal of the first two residues (Aspartate in position 1 and Alanine in position 2) by aminopeptidases, which leaves the glutamate at position 3 susceptible to spontaneous or enzyme‐catalyzed cyclization, forming AβpE3‐x.^92^ It has also been suggested that meprin β, besides Aβ2‐x, may also be involved in AβpE3‐x generation.^93^ This modification results in increased hydrophobicity, resistance to degradation and a more aggregation‐prone tendency that characterize AβpE3‐x species.^94^ As previously stated, the NT4X‐167 antibody recognized not only Aβ4‐4x species but also pyroglutamate AβpE3‐x species, showing epitope promiscuity.^86^ In addition, Crehan et al. reported two different AβpE3‐x monoclonal antibodies (07/01 IgG1 and 07/02 IgG2a) tested in aged APPSWE/PS1ΔE9 transgenic mice. Antibody 07/02, but not 07/01, showed cognitive improvement, better plaque clearance, and absence of microhemorrhage in vivo.^95^

### 8.3 Aβ5‐x

Aβ5‐x species, starting at Arginine 5, has been reported in AD brains, CAA cases as well as in transgenic mice models.^25^, ^81^, ^96^ The peptide production has been hypothesized as BACE1‐independent with potential involvement of α‐secretase‐like proteases (e.g., ADAM family proteases) or caspases.^96^, ^97^ Guzman et al. generated a novel monoclonal antibody (AB5‐3) specific to the N‐terminal end of Aβ5‐x, showing immunohistochemical evidence of Aβ5‐x in familial cases of AD (both vascular deposits and extracellular plaques) and its distribution in APP/PS1KI, 5XFAD, and 3xTG transgenic mouse models (extracellular plaque deposits without CAA).^98^ When tested on sporadic AD cases, AB5‐3 immunoreactivity was stronger in vascular deposits than in extracellular plaques, suggesting alternative mechanisms involving Aβ5‐x peptides depending on the AD onset.^98^

## 9 CONCLUSION, LIMITATIONS, AND FUTURE DIRECTIONS

Aβ4‐x is an important and sometimes underestimated player in AD. Aβ4‐x targeted immunotherapy in preclinical AD and vasculopathy studies proved to be a useful research tool for immunohistochemical and biochemical analyses of the properties and distribution of these species, leading to a better understanding of these neurological conditions. Although further studies will be required to confirm the safety and specificity of the reported antibodies for clinical studies, the extensive preclinical work on amyloid biology played a pivotal role in expanding the anti‐amyloid immunotherapy targeting Aβ N‐terminus.^12^, ^13^, ^14^, ^99^ First and second‐generation immunotherapies such as bapineuzumab (targeting amyloid residues 1–5) and antibody gantenerumab (targeting amyloid residues 3–11 and 18–27) reduced amyloid content by inducing receptor‐mediated microglial phagocytosis of the Aβ plaques.^80^, ^99^ However, their clinical outcomes were unsuccessful as none of them significantly improved cognitive decline, causing ARIA in the form of ARIA‐E (vasogenic edema) or ARIA‐H (microhemorrhages) as an adverse effect.^99^, ^100^, ^101^ To date, third‐generation antibodies targeting Aβ N‐terminus showed more promising results as the monoclonal antibodies lecanemab and donanemab are currently FDA approved for mild cognitive impairment and AD patients with elevated β‐amyloid in the brain.^99^, ^102^, ^103^ Lecanemab is a humanized IgG1 antibody that was developed to target the soluble Aβ protofibril conformation to promote its removal and reduce plaque formation.^104^ The antibody mediates amyloid clearance by activating microglial effector functions via its Fc fragment, showing a reduction in amyloid plaques and improvement in patients' cognitive decline.^102^, ^105^ Donanemab is a humanized immunoglobulin G1 monoclonal antibody that specifically targets N‐terminally truncated AβpE3‐x, predominantly presents within plaque cores.^106^ Additional studies reported that donanemab detected only a minor subset of plaques in AD and Down syndrome post‐mortem brains, although the antibody strongly detected the central core of the plaques.^107^ Results of the TRAILBLAZER‐ALZ 2 randomized clinical trial highlighted that donanemab treatment significantly slowed clinical progression at 76 weeks within participants with early symptomatic AD, amyloid, and tau pathology.^103^ Similar to previous therapies, ARIA was reported as adverse effects ranging from mild (headaches) to serious consequences (hospitalization in 1.6% of participants).^103^ Interestingly, the isotype of the antibodies used in clinical studies has shown a correlation with efficacy in CAA and ARIA‐related adverse effects. IgG1 antibodies anti‐Aβ (such as aducanumab, bapineuzumab, gantenerumab) have been associated with an increased risk of developing ARIA‐E and ARIA‐H due to microglia and complement activation.^108^ In support of this hypothesis, Crehan et al. reported that an IgG1 mutation (K322A) reducing the antibody binding to complement component 1q (C1q) prevented ARIA‐E in an AD mouse model with CAA.^95^ In addition, IgG2 and IgG4 anti‐Aβ antibodies (such as crenezumab) with a reduced effector function and lower Fcγ‐receptor‐mediated microglial activation, have shown reduced ARIA‐E incidence.^108^, ^109^ However, it must be noted that crenezumab did not show clinical efficacy.^109^ Based on these studies and clinical data available, it is possible to speculate that among the preclinical antibodies here discussed, those with IgG2 and IgG4 isotypes may have a better outcome in terms of ARIA induction compared to the IgG1 isotype. Although the reported results are encouraging, the preliminary stage that these antibodies are currently in does not give complete information on their safety profile or tolerability, which will require future in vivo work before transitioning to a clinical phase.

## AUTHOR CONTRIBUTIONS

Maria Luisa Valle conceptualized this review and wrote the manuscript. Donatello Arienzo reviewed and edited the manuscript. All authors read and approved the final version of the manuscript.

## CONFLICT OF INTEREST STATEMENT

The authors declare no conflicts of interest.

## ETHICS STATEMENT

Not applicable.

## Data Availability

Not applicable as no data was generated in this review.
